# High-Precision 3D-DIC Measurement Method Based on Improved Forward Newton Iteration

**DOI:** 10.3390/s23063317

**Published:** 2023-03-21

**Authors:** Huihui Wen, Ze Liu, Weizhe Gao, Yu Wang

**Affiliations:** School of Electrical Engineering, Hebei University of Science and Technology, Shijiazhuang 050018, China

**Keywords:** digital image correlation, three-dimensional measurement, Newton’s method

## Abstract

To solve the problems of the traditional 3D-DIC algorithm based on feature information or FFT search at the expense of accuracy in exchange for time, such as error-point extraction, mismatching of feature points, poor robustness, and accuracy loss caused by poor anti-noise performance, an improved high-precision 3D-DIC measurement method was proposed. In this method, the exact initial value is obtained by an exhaustive search. Then, the forward Newton iteration method is used for pixel classification, and the first-order nine-point interpolation is designed, which can quickly obtain the elements of Jacobian and Hazen matrix, and achieve accurate sub-pixel positioning. The experimental results show that the improved method has high accuracy, and its mean error and standard deviation stability and extreme value are better than similar algorithms. Compared with the traditional forward Newton method, the total iteration time of the improved forward Newton method is reduced in the subpixel iteration stage, and the computational efficiency is 3.8 times that of the traditional NR algorithm. The whole process of the proposed algorithm is simple and efficient, and it has application value in the precision occasions requiring high precision.

## 1. Introduction

3D digital image correlation (3D-DIC), as a reliable tool for full-field measurement of displacement and strain, provides an effective means for the measurement of displacement and strain in experimental mechanics, fracture mechanics, fatigue, and mechanical tests [1,2]. Compared with interferometry, 3D-DIC technology does not require a strict experimental setup and complex optical systems, so it has attracted a lot of attention. Surface deformation measurements can be easily performed using 3D-DIC technology because, in most cases, surface textures or artificial speckle processing can be used. The use of 3D-DIC has been accelerated in many disciplines, especially in the fields of mechanical engineering and material science, and provides the solution to multiple cross-scale scientific problems from macro, meso to micro [3], so it is of great significance for the study of improving the measurement accuracy of 3D-DIC.

The key technologies of the 3D-DIC method mainly include the following four aspects:

(1) Image acquisition: digital speckle images on the surface of the subject measured by the two cameras are acquired simultaneously;
(2) Deformation matching: find the corresponding point in the corresponding two speckle images;
(3) Camera parameter calibration: calibration of imaging model parameters of two cameras;
(4) 3D reconstruction: the 3D spatial coordinates of the corresponding point are calculated with the help of these parameters and the image coordinates of the corresponding point in the digital image.

Among them, deformation matching and 3D reconstruction are two key steps of 3D-DIC. In deformation matching and 3D reconstruction, the initial value estimation and sub-pixel registration algorithm are considered as the key techniques to improve the accuracy. Scholars at home and abroad have made many research attempts to improve and quantify the registration accuracy and computational efficiency of various algorithms [4,5,6]. To obtain the best value of the subpixel displacement field, nonlinear iterative algorithms such as the forward additive Newton–Raphson method (FA-NR), the forward additive Gauss-Newton method (FA-GN), Inverse compositional Gauss-Newton method (IC-GN), steepest descent, or Levenberg–Marquardt [7] can be used. FA-NR, FA-GN, and IC-GN are the most widely used DIC. The FA-NR algorithm was first proposed by Sutton [8], who introduced the Newton–Raphson method with differential correction to speed up the search time. Bruck [9] et al. completed the FA-NR algorithm by providing correction feedback for the six deformation parameters of the initially estimated first-order shape function. Lu and Gao [10,11,12] et al. introduced the second-order shape function to describe the surface deformation of objects, to improve the calculation accuracy of the displacement field and strain field of non-uniform deformation and improve the calculation accuracy of displacement. However, the second-order shape function is not the best choice in the case of high efficiency requirements, and the computation amount will appear redundant. In order to estimate the initial value of the region of interest quickly, Jiang and Zhang [13,14] used the fast-Fourier-transform-based cross correlation (FFT-CC) algorithm. Path-independent strategy has strong robustness to discontinuous deformation and high processing efficiency but is limited by the periodic continuation characteristics of FFT transform; it cannot deal with the large amplitude deformation field, such as rotational deformation, and the accuracy is still insufficient. Image feature is a very attractive method. A large number of key points and their description features can be obtained from image-feature extraction. These key points can be used for image registration. The registration process is based on feature vectors and has nothing to do with the spatial location of key points, which makes the registration method based on image features naturally suitable for large deformation scenes. In recent years, some scholars have introduced image feature methods into DIC initial value estimation algorithms [15,16,17,18,19], but image-feature extraction is time-consuming and has poor robustness. Therefore, high-precision 3D-DIC deformation matching and 3D reconstruction is still a challenging problem.

In the calculation process of the traditional 3D-DIC algorithm, the initial value acquisition method based on image features or FFT search is usually adopted, and the sub-pixel position gray value is obtained based on cubic spline interpolation algorithm. However, traditional methods based on the above mentioned methods will sacrifice accuracy for time, as well as error-point extraction, point-to-point mismatching, poor robustness, and anti-noise performance, resulting in accuracy loss, etc. To solve the above problems, this paper proposes a high-precision 3D-DIC measurement method with improved forward Newton iteration.

## 2. Key Technologies Related to 3D Digital Image

The 3D-DIC method combined with the DIC method and the principle of binocular stereo vision, using two alternating methods with a certain angle of a fixed camera surface-speckle image, and then through the image matching algorithm to calculate the image of the specific image coordinates, coupled with good calibration in advance two camera parameters and the relative position relation, calculates the three-dimensional coordinates on the surface of the object. The three-dimensional morphology of the object surface is obtained.

### 2.1. Related Matching Policy

There are two main matching strategies for 3D-DIC [20,21]: one is stereo matching of images taken by left and right cameras at the same time; the other is the deformation matching (correlation matching) of images before and after deformation taken by a single camera. In the first step, the corresponding points of the same object that point to the left and right images at a certain time can be found to reconstruct the 3D morphology of the object. The second step can track the position change of an object point in a single camera image during the deformation of the object under test. Combined with stereo matching and correlation matching, it can be used to solve 3D deformation parameters. In the 3D digital image correlation method in this study, the main image-matching method is gray matching method, which does not involve feature matching.

**Figure 1:**
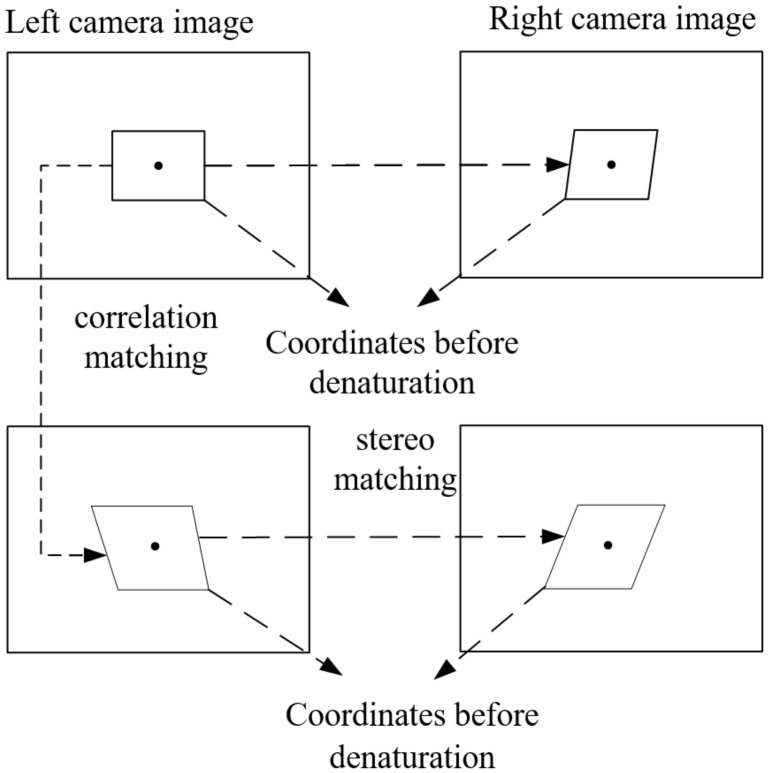
The matching strategy used in this article.

The 3D-DIC matching strategy used in this paper is shown in Figure 1, which involves two stereo matchings and one correlation matching. In the matching process of images collected by the left camera, the first image collected by the left camera is taken as the reference image. In the process of matching the left and right cameras, images collected by the left camera are used as reference images, and the matching calculation is made between each of the two images collected by the left and right cameras; then, the camera calibration parameters are used for 3D reconstruction.

### 2.2. Correlation Matching

Correlation matching is the most critical step of 3D-DIC, which is required in the key steps such as initial value estimation and sub-pixel calculation. In 3D-DIC, not only the center position of the left and right camera image subregion may change but also its shape may change. In this paper, the coordinates of corresponding points (x,y) and (x*, y*) in the subregion of the reference image and the target image are expressed as (ξ,η), the offset between the centers of the two matching subset patterns. The shape-mapping function of the whole reference subset and target subset can be expressed as

(1) $$\begin{array}{l} x^{*}=x+\xi +\xi_{x}(x-x_{0})+\xi_{y}(y-y_{0})\\ y^{*}=y+\eta +\eta_{x}(x-x_{0})+\eta_{y}(y-y_{0}) \end{array}$$

where *ξ* and *η* are the disparity of *P(x, y)* points in the center of the image subregion. *x* − *x*_0_ = Δ*x* and *y* − *y*_0_ = Δ*y* are the distance from point Q to the center of the subregion $\left(x^{*},y^{*}\right)$. The normalized least square distance function is defined to evaluate the similarity degree of the left and right camera image subregions or the image subregions before and after deformation, and its expression is as follows:

(2) $$C_{f,s}(w)=\sum_{x=-M}^{M}\sum_{y=-M}^{M}{\left[\frac{f(x,y)-f_{m}}{\sqrt{\sum_{x=-M}^{M}\sum_{y=-M}^{M}{\left[f(x,y)-f_{m}\right]}^{2}}}-\frac{s(x^{*},y^{*})-s_{m}}{\sqrt{\sum_{x=-M}^{M}\sum_{y=-M}^{M}{\left[s(x^{*},y^{*})-s_{m}\right]}^{2}}}\right]}^{2}$$

(3) $$\left\{\begin{array}{l} f_{m}=\frac{1}{{\left(2M\right)}^{2}}\sum_{x=-M}^{M}\sum_{y=-M}^{M}f(x,y)\\ s_{m}=\frac{1}{{\left(2M\right)}^{2}}\sum_{x=-M}^{M}\sum_{y=-M}^{M}s(x^{*},y^{*}) \end{array}\right.$$

where *f(x, y)* is the gray value of coordinate *(x, y)* in the image before deformation or the reference subarea of the left camera image. *f_m_* is the average grayscale of the image before deformation or the reference subarea of the left camera image. s(x*, y*) is the gray value of coordinates (x*, y*) in the target subarea of the deformed image or the right camera image. *s_m_* is the average gray of the target subregion. The pixel size of the subregion is 2*M ×* 2*M*.

## 3. Improved 3D-DIC Algorithm

### 3.1. Exhaustive Search Method

**Figure 2:**
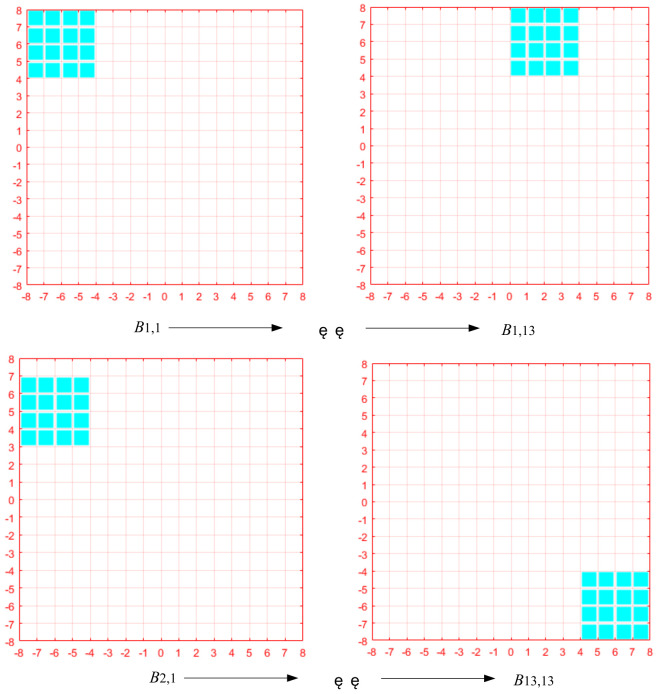
Exhaustive search method Search method.

The exhaustive search method is one of the most simple and accurate search methods, that is, a full search, which can ensure that the region with the highest global relevance is matched. The exhaustive search used in this paper includes search range and search path. Firstly, subregions are divided in the reference image, and then the search range of subregions is set. After the search range is set, the subregions are searched and matched point by point in the set range, and the matching criterion is ZNCC or ZNSSD. As shown in Figure 1, for example, the subregion is set as 4, and the search range is set as [−8, +8]. The subregion searches B_13,13_ from B_1,1_, along the path as shown in the Figure 2. The position of the most correlated subregion of ZNCC or ZNSSD is taken as the displacement calculation point, and its position and deformation are taken as the initial iteration value of the reference subregion. The advantage of this method is that it can accurately locate the real initial value of iteration by exhaustive search, and the matching operation process of each reference subregion is independent and does not depend on the matching results of other reference subregions. Therefore, in full field matching, all reference subregion operations can be executed in parallel, which is very convenient for parallel programming.

### 3.2. Forward Newton Iterative Method Based on First-Order Nine-Point Interpolation

Among digital image correlation methods, the traditional search method to obtain the maximum and minimum value of the correlation coefficient is relatively simple, but the calculation is time-consuming. By improving the correction term of the initial value, the forward Newton iteration method (FA-NR) takes the previously calculated integral pixel coordinates as the initial value for iterative calculation. After only a few calculations, the predetermined error range can be reached, which greatly improves the calculation speed compared with the thickness search method. There are six variables in the first-order function of subregion deformation:

(4) $$w=\left[u,v,\frac{\partial u}{\partial x},\frac{\partial v}{\partial y},\frac{\partial u}{\partial y},\frac{\partial v}{\partial x}\right]$$

Use $\Delta w=-H^{-1}g$ to represent the change of parameter w, where g is Jacobian matrix and H is Hessian matrix, which can be expressed as:

(5) $$g=\left[\frac{\partial s}{\partial w_{1}}\frac{\partial s}{\partial w_{2}}\frac{\partial s}{\partial w_{3}}\frac{\partial s}{\partial w_{4}}\frac{\partial s}{\partial w_{5}}\frac{\partial s}{\partial w_{6}}\right]$$

(6) $$H=\frac{\partial^{2}s}{\partial w_{i}\partial w_{j}}=\left[\begin{array}{cccc} \frac{\partial^{2}s}{\partial w_{1}{}^{2}} & \cdots & \cdots & \frac{\partial^{2}s}{\partial w_{1}\partial w_{6}}\\ \vdots & & & \vdots \\ \vdots & & & \vdots \\ \frac{\partial^{2}s}{\partial w_{6}\partial w_{1}} & \cdots & \cdots & \frac{\partial^{2}s}{\partial w_{6}{}^{2}} \end{array}\right]$$

**Figure 3:**
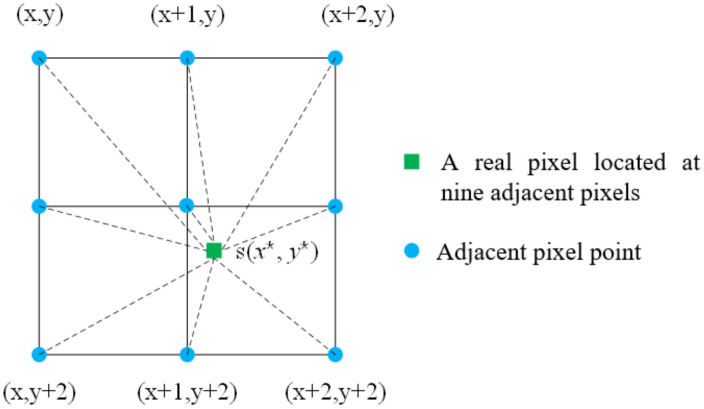
Schematic diagram of first-order nine-point interpolation.

It can be seen from the above equation that the first and second derivatives of the correlation coefficient s are the key to the problem. Generally, (x*, y*) is not an integer, and the gray value of (x*, y*) is obtained by gray interpolation of integer pixels. The diagram of first-order nine-point interpolation used in this paper is as follows (Figure 3).

In the figure above, the black dots represent actual integral pixel points (x and y are both integer values), and the green dots represent sub-pixel points whose pixel values can be interpolated from nine nearby pixels:

(7) $$s(x*,y*)=n_{1}+n_{2}x+n_{3}x^{2}y+n_{4}xy+n_{5}xy^{2}+n_{6}x^{2}+n_{7}y+n_{8}y^{2}+n_{9}x^{2}y^{2}$$

when the gray value of (x*, y*) is obtained, the elements of the Jacobi matrix and the Hazen matrix can be obtained by taking the partial derivative of s:

(8) $$\frac{\partial s}{\partial x*}=n_{2}+2n_{3}y+n_{4}y+n_{5}y^{2}+2a_{6}x+2n_{9}xy^{2}$$

(9) $$\frac{\partial s}{\partial y*}=n_{3}x^{2}+n_{4}x+2n_{5}xy+n_{7}+2n_{8}y+2a_{8}y+2n_{9}x^{2}y$$

The specific calculation process is as follows:

(1) The displacement value (u, v) is obtained by the exhaustive search method, which is taken as the initial value of the iteration

$$w=\left[u,v,\frac{\partial u}{\partial x},\frac{\partial v}{\partial y},\frac{\partial u}{\partial y},\frac{\partial v}{\partial x}\right]$$

(2) Gray interpolation is carried out on the search sub-region of the deformed image to obtain the gray value of the sub-pixel position, and the first-order nine-point interpolation is used.
(3) Find the Jacobian g and the Hazen H. Then, Formula (4.13) is used to solve the increment $\Delta w_{}^{\left(k\right)}$ and $w_{}^{\left(k+1\right)}$;
(4) To determine if the iteration is terminated: If $\|\Delta w_{}^{\left(k\right)}\|<\varepsilon$, terminate the iteration, otherwise continue with step (3), where ε indicates the allowable error of setting, which is generally set to 0.001. In addition, in order to prevent iteration from not converging, a maximum number of iterations (*N*_max_) should be set. When *k* = *N*_max_, the iteration should also be quit.

## 4. Experiment

**Figure 4:**
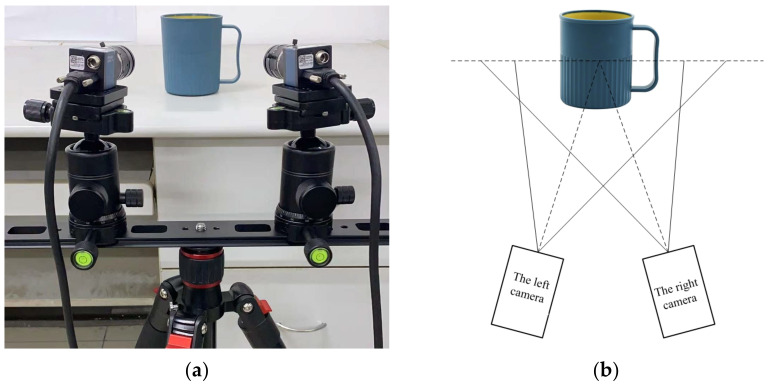
Experimental setup: (**a**) binocular system; (**b**) measurement diagram.

The experiments mainly include the 3D reconstruction experiments using the proposed algorithm and the subsequent 3D deformation measurement experiments. Figure 4 is the experimental device, in which Figure 4a is the binocular digital image correlation measurement system. The components of the binocular digital image correlation measurement system include the following equipment: two CCD cameras and lenses, a camera synchronization trigger device, an LED light source, a checkerboard calibration board, a triangle bracket, and a computer. Figure 4b shows the measurement schematic diagram.

In order to verify the measurement results of the proposed algorithm when the object changes dynamically, a three-dimensional rigid body displacement experiment is carried out. The NT501WML precision three-dimensional displacement platform is used to translate the measured object. The precision of the micro-displacement platform is 5 μm, so the accuracy of the displacement in the experiment can be guaranteed.

The experimental measurement steps are as follows:

(1) Spray a random speckle pattern on the surface of the measured object and fix it on the measuring platform.
(2) After assembling the camera and lens, install them on the triangular bracket and adjust the two cameras to a shooting Angle of about 45.
(3) The 3D deformation simulation measurement system was established, and the measured object was fixed on the micro-displacement platform. After adjusting the experimental devices, the dual-camera system was calibrated.
(4) Take a group of calibration plates in different positions and it save to the computer;
(5) Take a set of static pictures (only for 3D morphology measurement) or 3D deformation moving pictures (adjust the micro-displacement platform to make it appear 3D rigid body displacement to simulate 3D deformation) of the measured object and save it to the computer.
(6) The proposed algorithm is used for 3D reconstruction or 3D deformation calculation.

**Figure 5:**
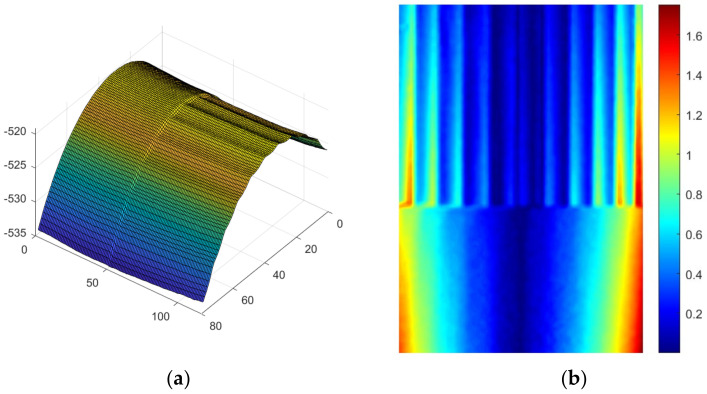
Experimental results: (**a**) 3D reconstruction result of the surface of the object under test; (**b**) 3D displacement measurement result of the rigid body on the surface of the object under test.

The obtained 3D reconstruction results are shown in Figure 5a. From the 3D surface reconstruction results, it can be seen that the characteristic shapes of each part of the measured object are correctly displayed, which verifies that the proposed algorithm can better achieve the 3D surface reconstruction of the measured object. A precision three-dimensional displacement table was used to rotate the measured object at a certain Angle. Figure 5b shows the displacement vectors in the three directions of X, Y, and Z after rotation. It can be seen from the displacement cloud diagram that the displacement conforms to the characteristics of rotation, that is, the displacement value near the center is small, and the displacement value at the left and right ends is large.

**Figure 6:**
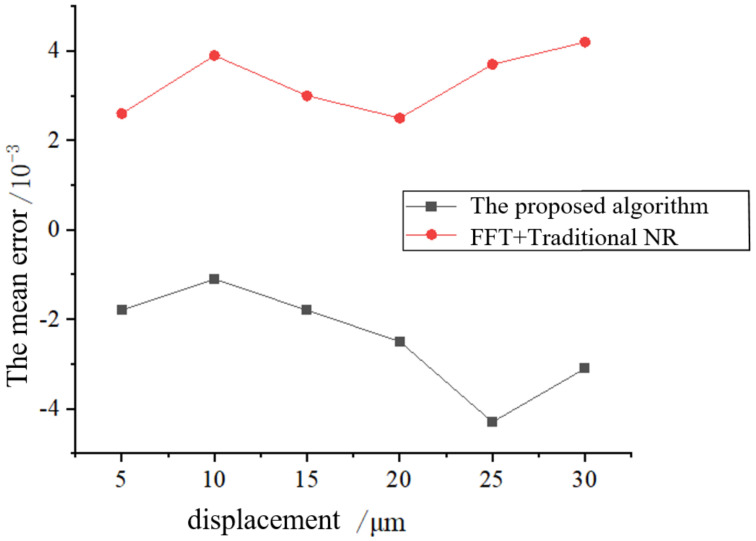
Mean error of different displacements.

In order to quantitatively verify the accuracy information of the proposed algorithm, a precision 3D displacement stage is used to translate 5 μm, 10 μm, …, 30 um along the *X*-axis, and the mean error was calculated using the proposed algorithm and FFT+ traditional NR algorithm, respectively. The results are shown in Figure 6. It can be seen that the sub-pixel displacement solution accuracy of the proposed algorithm is significantly higher than that of the FFT+ traditional NR algorithm. When the displacement is 25 μm, the mean error of the proposed method is 0.008 pixels higher than that of the FFT+ traditional NR algorithm.

In order to verify the efficiency of the proposed algorithm, Table 1 shows the time consuming information of subpixel displacement calculation in different computational subregions of the NR algorithm of traditional cubic spline interpolation and the proposed algorithm, and the convergence condition is Δw<ε=0.01. It can be seen that the sub-pixel phase time of the proposed algorithm is significantly lower than that of the traditional NR algorithm. Here, because the first-order interpolation used only requires nine-point fitting, the required accuracy can be achieved without the need for high-order invalid calculation. Moreover, the computational time of the proposed method only slightly increases with the increase in the subregion. The calculation speed of the traditional NR algorithm is 547 subregions/s, while the proposed method is 2122 subregions/s, which is 3.8 times of the traditional NR algorithm.

## 5. Conclusions

In this paper, an algorithm based on exhaustive search and first-order nine-point interpolation is proposed to solve the correlation of 3D digital images. The paper covers the following.

First, an exhaustive search is performed to obtain the initial value of the whole field. Then, the forward Newton iteration method is used for pixel classification, and the first-order nine-point interpolation is designed to quickly obtain the Jacobian and Hazen matrix elements and achieve accurate sub-pixel positioning. 

Second, in order to verify the effectiveness and solving accuracy of the algorithm, this paper first carries out 3D reconstruction experiments and 3D deformation simulation experiments, and then quantitative experiments from two aspects of accuracy and speed are conducted to compare the traditional FFT+NR algorithm with the algorithm in this paper.

The results show that the proposed algorithm has high computational accuracy, which is up to 0.008 pixels higher than the traditional FFT+NR algorithm. The subpixel time of the proposed algorithm is significantly lower than that of the traditional NR algorithm, and the computation speed is 3.8 times that of the traditional NR algorithm.

## Figures and Tables

**Table 1:** Comparison of calculation time.

Area Size	Traditional NR Algorithm/s	The Proposed Algorithm/s	Count the Total Number of Subregions
31 × 31	4.382	1.131	2401
41 × 41	5.231	1.652	
51 × 51	7.982	2.012	

## Data Availability

Not applicable.
